# Relevant Properties and Potential Applications of Sericin in Bone Regeneration

**DOI:** 10.3390/cimb45080426

**Published:** 2023-08-15

**Authors:** Jwa-Young Kim, Seong-Gon Kim, Umberto Garagiola

**Affiliations:** 1 Department of Oral and Maxillofacial Surgery, Hallym University Kangnam Sacred Heart Hospital, Hallym University Medical Center, Seoul 07441, Republic of Korea; jwayoung@hanmail.net; 2 Department of Oral and Maxillofacial Surgery, College of Dentistry, Gangneung-Wonju National University, Gangneung 28644, Republic of Korea; 3 Biomedical, Surgical and Oral Sciences Department, Maxillofacial and Dental Unit, School of Dentistry, University of Milan, 20122 Milan, Italy; umberto.garagiola@unimi.it

**Keywords:** sericin, bone graft applications, biocompatibility, immune responses, tissue engineering, bone healing

## Abstract

The potential of sericin, a protein derived from silkworms, is explored in bone graft applications. Sericin’s biocompatibility, hydrophilic nature, and cost-effectiveness make it a promising candidate for enhancing traditional graft materials. Its antioxidant, anti-inflammatory, and UV-resistant properties contribute to a healthier bone-healing environment, and its incorporation into 3D-printed grafts could lead to personalized medical solutions. However, despite these promising attributes, there are still gaps in our understanding. The precise mechanism through which sericin influences bone cell growth and healing is not fully understood, and more comprehensive clinical trials are needed to confirm its long-term biocompatibility in humans. Furthermore, the best methods for incorporating sericin into existing graft materials are still under investigation, and potential allergic reactions or immune responses to sericin need further study.

## 1. Introduction

Bone grafting is a surgical procedure that is designed to fix problems associated with bones or joints. This operation plays an indispensable role in various medical fields, ranging from orthopedics, dental procedures, and plastic surgery to treatments of trauma, disease, or skeletal abnormalities. The procedure traditionally involves the transplantation of bone tissue, known as a graft, to repair and rebuild diseased or damaged bones [1]. A bone graft can fill a void where bone is absent, providing a scaffold upon which new bone can grow [2]. This not only stimulates bone growth but also aids in maintaining bone strength and structure [3]. Therefore, bone grafts hold immense importance in healing complex fractures, aiding bone growth around surgically implanted devices, and promoting fusion between bony fragments [4].

Traditionally, the sources of bone graft materials have been broadly classified into four categories: autografts (from the patient’s own body), allografts (from a human donor), xenografts (from a nonhuman species), and synthetic substitutes [5]. Autografts, derived from the patient’s own body, are considered the “gold standard” for bone grafts due to their high biocompatibility and osteoinductive properties [6]. However, the harvesting procedure may lead to donor site morbidity, and there are limitations on the available volume. Allografts and xenografts, obtained from human donors and animal species, respectively, bypass the issue of limited material availability [7,8,9]. However, they carry risks of immunogenicity, disease transmission, and may lack the osteoinductive properties found in autografts. Synthetic substitutes, on the other hand, such as hydroxyapatite, bioactive glasses, and polymers, can be manufactured in large quantities and tailored to specific needs [10,11]. Nonetheless, these substitutes may not fully replicate the biological properties of natural bone, potentially affecting graft success. Thus, while each of these traditional materials has played a significant role in bone grafting, they also come with their own set of limitations. These issues highlight the need for exploring new materials for bone grafts, ones that can combine the benefits of the traditional materials while overcoming their shortcomings [12].

Sericin is a natural protein primarily derived from the cocoon of the *Bombyx mori* silkworm, an insect that has been at the heart of silk production for thousands of years [13]. It is secreted by the silkworm during the spinning of its cocoon and is used as a “bonding” agent, accounting for approximately 20–30% of the total weight of the silk cocoon [14]. Additionally, spiders also produce sericin [15].

What makes sericin unique is its “glue-like” characteristic, which allows it to bind the two fibroin filaments together, forming a single silk thread [16]. This feature comes from its molecular structure, which is characterized by its high content of polar amino acids, including serine, aspartic acid, and threonine [17,18]. These polar amino acids form hydrogen bonds with the surrounding water molecules, leading to its adhesive and gel-forming properties [19].

**Figure 1:**
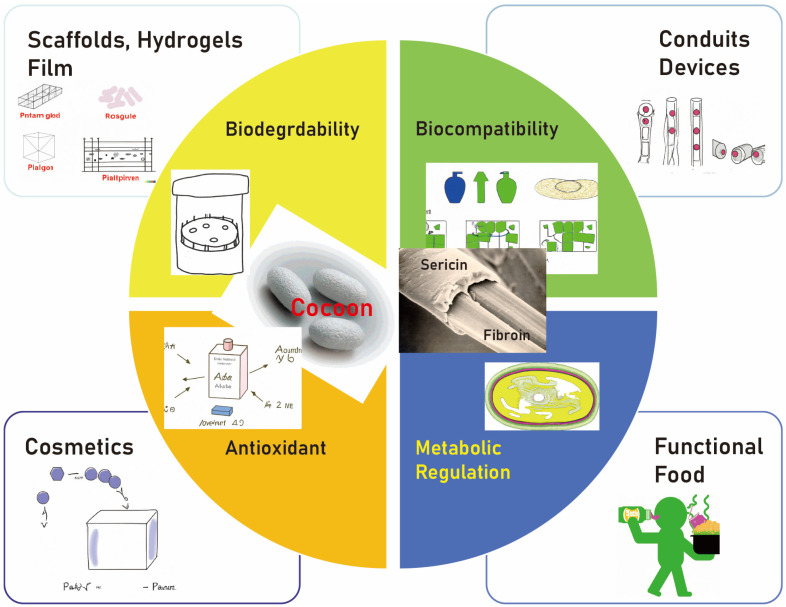
The biomedical field is increasingly recognizing sericin for its outstanding biocompatibility and biodegradability. These attributes position sericin as a potential candidate for diverse biomedical uses, such as tissue engineering and drug delivery.

In addition to its bonding capabilities, sericin has gained attention in the biomedical field for its exceptional biocompatibility and biodegradability [17,20]. Biocompatibility refers to the ability of a material to perform its desired function, without eliciting any undesirable local or systemic effects in the host, while biodegradability ensures that the material can be broken down into harmless products by biological systems [20]. These characteristics make sericin a promising material for various biomedical applications, including tissue engineering and drug delivery (Figure 1).

Moreover, sericin is also known for its antioxidant, UV-resistant, and antibacterial properties [17]. The antioxidant property of sericin helps in neutralizing harmful free radicals in the body, thereby preventing cell damage [21]. Its UV-resistant property provides protection against ultraviolet rays, making it useful in the cosmetics industry [22]. Additionally, its antibacterial property inhibits the growth of certain bacteria, providing potential applications in wound healing [23]. Given these unique properties, sericin’s application in the field of bone grafting presents an intriguing direction for future research.

## 2. Sericin Properties Relevant to Bone Grafting

The biocompatibility of sericin, which is derived from the *B. mori* silkworm cocoon, has been extensively investigated in several studies [15,16,17]. As a protein, sericin possesses inherent affinity with bodily tissues, thereby reducing the likelihood of any detrimental immune responses upon its introduction into the body [24]. This characteristic renders sericin an extremely promising candidate for various biomedical applications, encompassing drug delivery, tissue engineering, and wound healing [25].

The unique properties of sericin have been the subject of scientific exploration due to its potential in biomedical research. It has been found that sericin displays a remarkable ability to integrate with bodily tissues, owing to its natural composition and structure [17]. This integration not only minimizes any potential adverse immune reactions but also ensures a more seamless and efficient interaction between the material and the host organism [24].

The biocompatibility of sericin is of particular interest in the field of drug delivery. Sericin-based carriers have been developed to encapsulate and transport various therapeutic agents, such as drugs and biologics, to target sites within the body [25]. The compatibility of sericin with bodily tissues promotes the controlled release of these agents, enhancing their therapeutic efficacy while reducing the risk of adverse effects [17]. Furthermore, sericin’s ability to interact with cells at the molecular level allows for targeted drug delivery, enabling precise localization of therapeutic agents to specific tissues or cells [24].

Sericin’s potential for tissue engineering applications is also noteworthy. Its biocompatibility and affinity with bodily tissues make sericin an ideal material for constructing scaffolds that support cell growth and tissue regeneration [25]. By providing a conducive environment for cell attachment, proliferation, and differentiation, sericin-based scaffolds can facilitate the regeneration of damaged or diseased tissues [17]. Additionally, sericin’s ability to modulate cellular behavior and promote angiogenesis further enhances its suitability for tissue engineering applications [24].

Wound healing is another area where sericin’s properties can be harnessed. The biocompatibility and affinity of sericin with bodily tissues promote wound healing by facilitating the formation of a favorable microenvironment for tissue regeneration [25]. Sericin-based dressings have been shown to accelerate wound closure, reduce scar formation, and enhance tissue regeneration [21]. Furthermore, sericin’s antimicrobial properties contribute to the prevention of infection, a common complication in wound healing [23].

In the field of bone grafting, the biocompatibility of the graft material is of utmost importance. It is crucial for the material to not only interact safely with the bone tissues but also facilitate the growth and development of new bone cells. Research has indicated that sericin, owing to its proteinaceous nature and high biocompatibility, shows promise in promoting cellular adhesion and proliferation [24,26]. This makes it an excellent candidate for bone graft applications. Additionally, sericin is biodegradable [20], which means that it can be gradually broken down and replaced by the patient’s own cells. This feature further reduces the risk of long-term foreign body reactions.

This sets sericin apart from many synthetic materials commonly used in bone grafting, as these materials may persist in the body and lead to chronic inflammation or other complications. By contrast, sericin’s biodegradability ensures a more seamless integration with the surrounding tissues, minimizing potential adverse effects. Further research is needed to fully explore the potential of sericin in bone grafting applications. However, the preliminary findings regarding its biocompatibility and ability to promote cellular adhesion and proliferation are promising [24,26]. If these initial results are confirmed and expanded upon, sericin has the potential to revolutionize the field of bone grafting by providing a safer and more effective alternative to synthetic materials.

Sericin, a protein derived from the *Antheraea pernyi* silkworm, has been found to have a significant impact on the formation of hydroxyapatite (HAp) nanoneedle-like crystals [27]. This process is influenced by the concentration of sericin as well as the length of time allowed for mineralization [28]. In vitro assays have demonstrated that mineralized sericin/HAp composites exhibit a higher level of osteogenic differentiation in bone marrow stem cells (BMSCs) compared to nonmineralized composites [28]. One of the key mechanisms by which sericin promotes this mineralization process is through its stimulation of collagen production. Collagen is a crucial protein in the composition of the bone matrix [29]. Sericin’s ability to enhance collagen production is thought to play a significant role in facilitating the formation and growth of HAp crystals. Furthermore, the amino acids present in sericin, particularly glutamic acid and aspartic acid, have been identified as key players in regulating the formation and growth of HAp crystals [30,31]. These amino acids have the unique ability to attract calcium and phosphate ions, thereby increasing the local supersaturation and creating an environment conducive to the nucleation and growth of HAp crystals. The findings regarding sericin’s ability to promote the formation of HAp crystals and enhance osteogenic differentiation in BMSCs hold great promise for the field of bone grafting. The potential of sericin as a biomaterial in bone graft applications should be further explored through rigorous research and experimentation. If confirmed, sericin could revolutionize the field by providing a biocompatible and effective alternative to existing synthetic materials.

The use of sericin-based biomaterials, such as sericin-coated titanium and sericin nanofiber, has shown promising results in promoting osseointegration [32] and osteogenic differentiation [28]. In a study, it was observed that sericin extract from *B. mori*, when applied at a concentration of 40 μg/mL, significantly increased the proliferation of osteoblast cells by up to 135% compared to the untreated control [33]. Furthermore, it was found that the unmodified form of sericin possesses dual-functional properties, displaying both cytocompatibility towards osteoblast cells and antibacterial/biofilm activity against *Staphylococcus aureus* [33]. These significant findings have paved the way for the development of biomaterials based on sericin for bone tissue engineering applications.

## 3. Studies on Sericin in Bone Grafting

**Figure 2:**
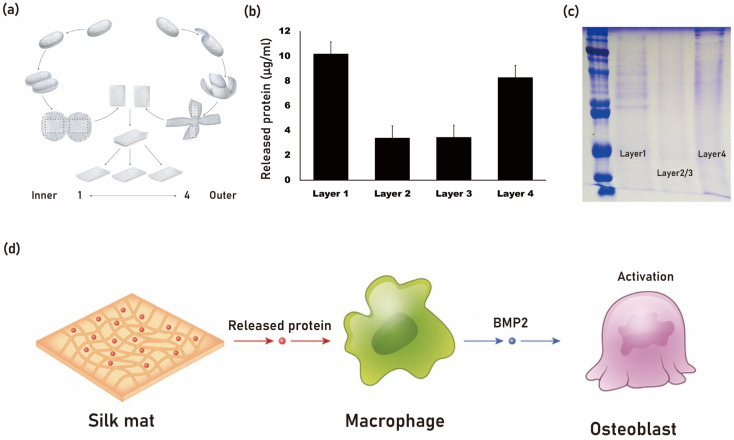
The silk mat, derived from silkworm cocoon (**a**), exhibits a varied protein composition from its inner to outer layers. The innermost (layer 1) and outermost layers (layer 4) release a higher amount of protein (**b**). When the silk mat is immersed in phosphate-buffered saline (PBS), a soluble protein fraction is released, as depicted in the SDS page gel (**c**). It is theorized that a protein present in the solution may induce macrophages to release BMP-2, which can potentially enhance bone regeneration by stimulating osteoblasts (**d**).

Silk mat, derived from the silkworm cocoon of *B. mori*, contains various proteins, including sericin [34]. It has been observed that when silk mat is used as a membrane for guided bone regeneration, it exhibits comparable new bone formation to that of collagen membrane [35]. When silk mat is placed in phosphate-buffered saline (PBS), a soluble fraction is released into the saline (Figure 2). This solution, after protein concentration determination, is applied to macrophages to examine differentially expressed genes using cDNA microarray analysis [36]. Among the genes studied, bone morphogenetic protein-2 (BMP-2) has shown increased expression upon administration of the silk mat-derived solution [36]. BMP-2 is a potent osteogenic protein [37]. It is hypothesized that a protein present in this solution may stimulate macrophages to secrete BMP-2. Isolation of this protein could potentially be advantageous for bone regeneration purposes.

**Figure 3:**
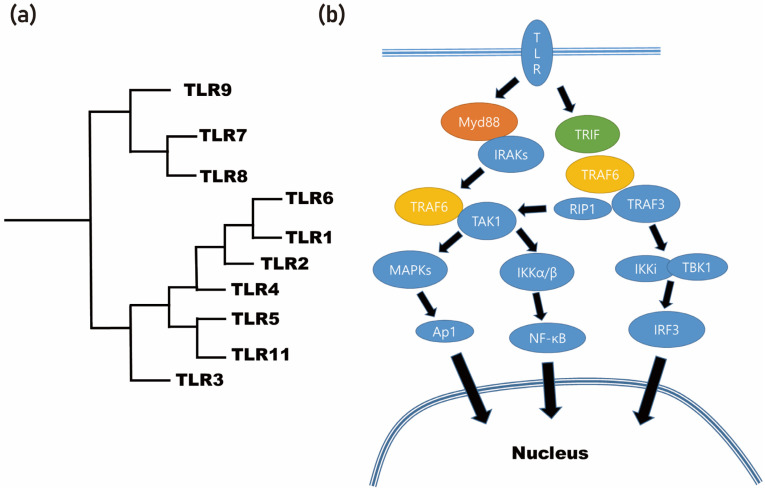
Foreign proteins often elicit immune responses through the recognition of Toll-like receptors (TLRs) by immune cells. (**a**) Phylogenetic analysis of TLR. (**b**) Summarized signaling pathway of Toll-like receptor (MyD88: myeloid differentiation primary response 88; TRIF: toll/interleukin-1 receptor (TIR) domain-containing adapter-inducing interferon-β; IRAKs: interleukin-1 receptor-associated kinases; TRAF6: tumor necrosis factor (TNF) receptor-associated factor; TAK1: TRAF6-induced transforming growth factor beta-activated kinase 1; MAPKs: mitogen-activated protein kinases; NF-κB: nuclear factor-κB; ERK1/2: extracellular signal-regulated kinase 1/2; JNK: c-Jun N-terminal kinase; AP-1: activator protein-1; RIP1: receptor-interacting serine/threonine protein kinase 1; IKKi: IκB kinase-I; TBK1: TRAF family member-associated NF-κB activator (TANK)-binding kinase 1; IRF3: interferon regulatory transcription factor 3).

Through the application of different degumming temperatures, it is possible to obtain differential protein profiles from the silk cocoon [38]. By utilizing a molecular weight-based filter, these proteins can be classified into detailed groups [38]. As a result of this process, the BMP-2-inducing protein present in the silk mat was identified as sericin [38]. Sericin, being an insect-derived protein, is considered a foreign protein for mammals [39]. Foreign proteins often elicit immune responses through the recognition of pattern-recognizing receptors, such as Toll-like receptors (TLRs), by immune cells (Figure 3). In the case of sericin, when it is applied to macrophages, it is recognized by the TLRs present on these immune cells [38,39].

Consequently, the BMP-2-inducing effect of sericin is mediated by the TLRs expressed on macrophages [39]. It is important to note that TLRs are pattern-recognizing receptors, and therefore, the conformation of sericin protein could potentially influence the expression level of BMP-2 [38,40]. This finding regarding the role of sericin in inducing BMP-2 expression sheds light on the potential mechanisms underlying the bone regenerative properties of silk mat [36]. Understanding the interaction between sericin and TLRs provides valuable insights for the development of novel approaches for bone regeneration purposes.

**Figure 4:**
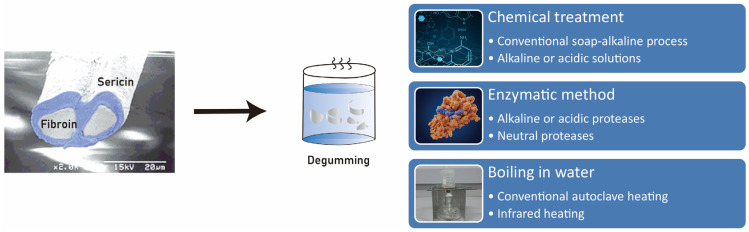
The “degumming process” refers to the removal of sericin from the silkworm cocoon. There are various types of degumming processes. This process is critical as it significantly influences the conformation of sericin.

The degumming process plays a crucial role in influencing the conformation of sericin, a protein derived from silk (Figure 4). It has been observed that using a degumming temperature below 65 ºC results in a greater abundance of β-sheet conformation in sericin compared to using temperatures above 100 °C [38,41]. Drying sericin may extend its storage time, and β-sheet crystallization also increases with decreasing temperature [41]. It has been found that sericin with a rich β-sheet conformation exhibits a higher ability to induce the expression of BMP-2 in macrophages, compared to sericin with other conformations [40].

However, it is important to note that the low-temperature method of degumming has been found to have a lower yielding ratio compared to the high-temperature method [40]. This limitation can be overcome by employing alternative degumming solutions to produce sericin with a high content of β-sheet conformation. For example, studies have demonstrated that using a solution of 70% ethanol [18], formic acid [18], or 2% 4-hexylresorcinol (4HR) [40] can stabilize the protein at high temperatures and yield a greater amount of β-sheet rich sericin, in comparison to using distilled water. These alternative degumming solutions have shown promising results in promoting the formation of β-sheet structures in sericin. Ethanol, formic acid, and 4HR have been found to effectively stabilize the protein during the degumming process, leading to a higher content of β-sheet conformation. This is crucial, as the β-sheet structure is associated with the superior mechanical properties and stability of sericin-based materials.

The use of ethanol as a degumming solution has been reported to enhance the formation of β-sheet structures in sericin [18]. Ethanol acts as a denaturant, disrupting the non-covalent interactions and hydrogen bonds in the protein, promoting the rearrangement of the protein chains into β-sheet structures. This results in a higher content of β-sheet conformation in the extracted sericin. Similarly, formic acid has been shown to have a stabilizing effect on sericin, promoting the formation of β-sheet structures [18]. Formic acid acts as a chemical denaturant, altering the secondary structure of the protein and facilitating the rearrangement of the protein chains into β-sheet conformations. This leads to an increased content of β-sheet structures in the sericin extract. Another alternative degumming solution, 4HR, has been found to effectively stabilize sericin at high temperatures and promote the formation of β-sheet structures [40]. 4HR acts as a chemical chaperone, interacting with the protein molecules and facilitating the rearrangement of the protein chains into β-sheet conformations [42]. This results in a higher yield of β-sheet rich sericin.

**Figure 5:**
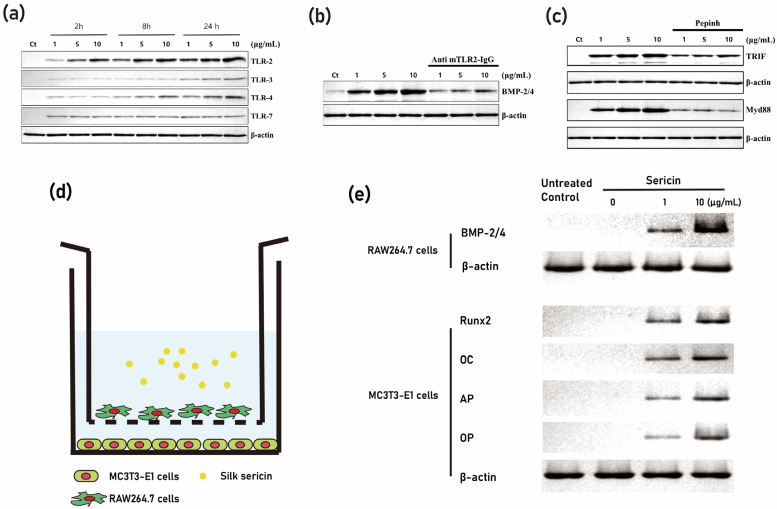
Activation of the TLR-mediated pathway through the application of sericin. (**a**) demonstrates increased expression levels of TLR-2, TLR-3, and TLR-4 induced by sericin. (**b**) shows that the application of mouse TLR-2 neutralizing antibodies results in a decrease in the level of BMP-2/4 expression initiated by sericin. (**c**) reflects that the application of an inhibitory peptide (Pepinh) for TRIF and MyD88 yields a similar effect to that of TLR-2 antibodies. A coculture experiment with RAW264.7 cells (macrophage) and MC3T3-E1 cells (osteoblast) supports these findings. (**d**) provides a schematic representation of the coculture system. (**e**) reveals that sericin application boosts BMP-2/4 levels in RAW264.7 cells. In MC3T3-E1 cells, the expression levels of Runx2, osteocalcin, and osteonectin were found to be higher in the RAW264.7 cells of the sericin application group compared to the RAW264.7 cells not treated with sericin (modified from author’s own publication [38]).

Immunomodulation is a tissue engineering technique that involves the use of molecules to manipulate the immune response in a desired direction [43]. In the context of bone tissue regeneration, bone resident macrophages play a crucial role [44]. It has been observed that sericin, a protein derived from silk, can induce the expression of BMP-2 in macrophages [38]. This is important because BMP-2, when derived from macrophages, can stimulate resting osteoblasts, and activate them (Figure 5). In coculture experiments, the administration of sericin to macrophages was shown to activate osteoblasts [38]. Furthermore, when a sericin-incorporated gelatin sponge is implanted into a bony defect, there is a higher level of new bone formation compared to when using a gelatin sponge alone [38,40]. Additionally, the expression level of BMP-2 is higher in the tissue of the sericin-incorporated gelatin sponge group compared to the other groups [38].

*B. mori*, commonly known as the silkworm, encompasses various subspecies, and these subspecies may exhibit differences in the amino acid sequence of sericin, a protein derived from silk. In a comparative analysis of three different subspecies, namely *Baegokjam, Yeonnokjam*, and *Goldensilk*, it was found that the expression level of BMP-2, an important factor in bone regeneration, was highest in the *Baegokjam* subspecies [45]. *Sericinjam* is a genetically modified silkworm that has been selectively bred to produce sericin exclusively [46]. This selective breeding allows sericin to be obtained without the need for the traditional degumming process [47]. However, it should be noted that natural sericin obtained from *Sericinjam* cannot be directly grafted into the body without undergoing a cleaning process. Therefore, for the application of sericin from *Sericinjam* as a bone graft material, special processing techniques are required to ensure its effectiveness.

These findings emphasize the intricate nature of sericin and underscore the significance of comprehending its specific characteristics and processing requirements for its use as a bone graft material. Further investigation is warranted to elucidate the underlying mechanisms responsible for the differential expression of BMP-2 among *B. mori* subspecies and to optimize the processing techniques for sericin derived from *Sericinjam*. In particular, the relationship between TLRs and BMP-2 remains unclear. It has been observed that other ligands for TLRs can activate osteoblasts [48]. Conversely, when a TLR inhibitor is applied to grafts, it inhibits bone regeneration [49]. However, the activation of TLRs by microorganisms generally promotes bone resorption through osteoblast-mediated osteoclastogenesis [48]. Models deficient in TLRs exhibit reduced bone resorption [50]. When foreign materials bind to TLRs, various types of heterodimers can be formed based on the different conformations of foreign proteins. Some of these heterodimers can activate bone resorption [51]. While TLR2 is known to play a crucial role in sericin [18], the detailed mechanisms involved are still under investigation.

In our previous study, we investigated the use of 4HR as an incorporating agent of sericin to increase the β-sheet structure [40]. Notably, 4HR has been shown to increase the expression level of the transforming growth factor-β family including BMP-2 through epigenetic regulation [52,53]. Therefore, the elevated expression level of BMP-2 observed with 4HR-incorporated sericin could be attributed to the release of 4HR from the graft or a conformational change in sericin. To determine whether the increased expression of BMP-2 by 4HR-incorporated sericin is mediated by free 4HR or the conformational change of sericin, we examined the effect of inhibiting TLRs. If the increased expression of BMP-2 is solely mediated by free 4HR, the inhibiting TLRs do have any effect on the expression level of BMP-2 from 4HR-incorporated sericin. However, our findings showed that the inhibition of TLRs decreased the expression level of BMP-2 by 4HR-incorporated sericin [40]. This suggests that the conformational change of sericin, rather than the presence of free 4HR, is responsible for the increased expression of BMP-2.

## 4. Comparative Analysis

The use of silk mat as a sericin conjugated graft has been investigated in several studies. In a rat calvarial defect model, silk mat demonstrated superior bone regeneration compared to expanded polytetrafluoroethylene (ePTFE) [35]. Furthermore, clinical trials evaluating silk mat as a bone graft material have shown promising results. In a split mouth model, the bone regeneration achieved with silk mat was comparable to that of high-density PTFE [54]. Additionally, when compared to a control group without any graft, the silk mat group exhibited significantly higher levels of bone regeneration [55].

When considering the use of sericin as a bone graft material, it is of utmost importance to carefully select an appropriate scaffold. This selection is crucial, as the scaffold plays a critical role in supporting the growth and regeneration of new bone tissue. In a comparative study between gelatin and collagen scaffolds, it was found that collagen scaffolds demonstrated significantly higher levels of bone regeneration when combined with sericin grafts [56]. These findings underscore the potential of silk mat as a sericin-conjugated graft for promoting bone regeneration.

However, it is worth noting that further research is necessary to optimize fabrication techniques and scaffold design to enhance the efficacy of sericin-based bone graft materials. This research should aim to develop improved methods for incorporating sericin into the scaffold matrix, ensuring its proper distribution and retention within the scaffold structure. Additionally, the mechanical properties of the scaffold must be carefully considered. The scaffold should possess adequate strength and stability to withstand the physiological forces exerted on it during the healing process. Moreover, the scaffold should provide a suitable microenvironment for cell adhesion, proliferation, and differentiation. Furthermore, the bioactive properties of sericin should be further investigated to better understand its mechanisms of action in promoting bone regeneration. It is important to elucidate the specific interactions between sericin and bone cells, as well as the signaling pathways involved in the osteogenic differentiation process. This knowledge will contribute to the development of more targeted and effective sericin-based graft materials.

The potential of sericin lies in its ability to act as a scaffold for bone regeneration, while also providing osteoinduction and immunomodulatory activities. This biopolymer is now being studied in a variety of composite scaffold forms that can not only fill bone defects but also stimulate natural bone regeneration. Sericin-based hydrogel scaffold demonstrates excellent biocompatibility, aids cell adhesion and proliferation, and promotes osteogenesis [57]. Implanted in rat calvarial defects, the scaffold facilitated new bone regeneration within 12 weeks by inducing autologous bone marrow-derived mesenchymal stem cell differentiation [57]. Further research by Fu et al. [58] explored the combination of sericin with nanohydroxyapatite and graphene oxide in an alginate-based nanocomposite hydrogel. This scaffold not only promotes osteogenesis but also creates a favorable osteoimmune microenvironment by inducing M2 macrophage polarization [58]. The synergistic effect of immunomodulation and osteoinduction exhibited by this scaffold leads to effective bone regeneration [58].

In addition to the combination with synthetic materials, sericin also has shown potential in mineralization applications. Studies on the mineralization of *A. pernyi* silk sericin found the protein capable of facilitating HAp crystal nucleation, which is a vital aspect of bone formation. Both Yang et al. [59] and Jiayao et al. [60] confirmed that the *A. pernyi* sericin can stimulate cell adhesion, proliferation, and osteogenic differentiation of BMSCs. Griffanti et al. [61] explored the use of sericin in silk sericin-functionalized dense collagen-fibrin hybrid hydrogels. They found that the addition of sericin accelerated mineralization and improved the proliferation and osteoblastic differentiation of seeded MC3T3-E1 preosteoblastic cells, offering a new approach to designing scaffolds that mimic bone ECM. Finally, investigations into the degradation pattern of porous hydroxyapatite microspheres created in the presence of sericin found that these materials degrade at a rate that matches new bone formation, a crucial attribute for any biomaterial intended for bone graft applications [62].

The development of sericin-based scaffolds offers a promising alternative for bone grafts. Whether used in combination with other materials or leveraged for its mineralization properties, sericin provides scaffolds with improved osteoinduction, cell adhesion, and proliferation. While these early studies show promise, further research is needed to fully understand the mechanisms at play and to advance these technologies towards clinical application.

## 5. Potential Applications and Future Directions

Sericin’s potential in bone graft applications is under active exploration and brings a new horizon to the world of tissue engineering. Numerous studies suggest that the addition of sericin to traditional graft materials might enhance the graft’s biocompatibility and foster the growth of bone cells [25,28]. Sericin’s hydrophilic nature allows for better cell adhesion and proliferation, making it an excellent candidate for bone grafts [17]. Its natural abundance and biodegradability make it a cost-effective and sustainable choice [21]. There is also research suggesting sericin can accelerate bone healing by enhancing calcium deposition and collagen formation, both critical components in bone remodeling [27].

In addition to these, the antioxidant [21], anti-inflammatory [63], and UV-resistant [22] properties of sericin contribute to a healthier bone-healing environment. Antioxidant and anti-inflammatory properties can reduce damage from oxidative stress and inflammation, common issues in post-surgery recoveries [21,63]. Its UV-resistant property may protect cells from harmful radiation during certain diagnostic procedures [22]. Moreover, incorporating sericin into 3D-printed grafts is a promising avenue for personalized medicine [64]. These 3D-printed grafts could potentially be customized to patients’ specific needs, making surgeries more effective and recovery more efficient.

Three-dimensional printing, also known as additive manufacturing, enables the fabrication of complex structures with precise control over the spatial distribution of cells and biomaterials. Silk protein, particularly fibroin and sericin, has garnered significant attention in tissue engineering due to its exceptional biocompatibility, biodegradability, and mechanical properties. This paper delves into the unique properties of sericin that make it an attractive bioink for 3D printing in tissue engineering applications.

Sericin can be extracted from silk through clean degumming processes. Studies have demonstrated the successful grafting of sericin with glycidyl methacrylate (GMA) to improve its stability and printability as a bioink [65]. The resulting sericin-based bioinks have shown promising characteristics, such as enhanced mechanical strength, thermal stability, and resistance to protease hydrolysis. These attributes make sericin an ideal candidate for fabricating 3D scaffolds with improved mechanical integrity and tailored degradation properties [66]. The combination of sericin-based bioinks and 3D printing technology has led to the development of diverse scaffolds for tissue engineering [67]. Bioprinted sericin scaffolds have demonstrated improved swelling properties and sustained drug release capabilities, making them suitable for drug delivery applications [68]. Moreover, these scaffolds have shown excellent cytocompatibility, facilitating cell adhesion, proliferation, and tissue formation [69].

Sericin’s unique properties have been harnessed in creating skin substitutes for wound healing. As an essential component of the epidermal layer, 3D-printed sericin-based constructs have shown promise in mimicking the features of native skin, providing a protective barrier against dehydration and external hazards [64]. These constructs have exhibited excellent mechanical properties, wettability, antimicrobial activity, and cytocompatibility, making them attractive candidates for skin tissue regeneration [64]. While the combination of sericin and 3D printing in tissue engineering has shown tremendous potential, there remain areas for further exploration and improvement. Advancements in bioprinting techniques, such as multimaterial printing and vascularization, hold promise in enhancing the complexity and functionality of sericin-based constructs [70]. Additionally, investigations into using sericin in combination with other biomaterials may yield synergistic effects and broaden the scope of applications in tissue engineering [68].

Sericin’s integration into 3D printing as a bioink presents an exciting avenue for tissue engineering applications. Its unique properties, coupled with the versatility of 3D printing, offer novel possibilities in regenerative medicine, drug delivery, and wound healing. The current progress indicates that sericin-based bioinks have the potential to revolutionize tissue engineering strategies and contribute to the development of innovative and patient-specific biomedical solutions.

Sericin-based nanoformulation can be used in the medical field [71]. There are several methods for the preparation of sericin-based nanoparticles. Desolvation is a technique that employs a desolvating agent like acetone or ethanol to dehydrate proteins in a water-based solution, resulting in a coiled protein conformation [72]. A double desolvation method can be used to create thinner, smaller nanoparticles [72]. Crosslinking of protein amino groups can produce denser and more stable nanoparticles [73]. However, drawbacks include the use of organic solvents and potentially toxic crosslinkers, such as glutaraldehyde, and low encapsulation efficiency [74,75]. Self-assembly is a method that results in functional complexes through the weak noncovalent interactions of small building blocks [76]. Each of these techniques can be employed to create sericin-based nanoparticles with different properties and applications. For example, desolvation has been used to create nanoparticles that enhance the growth inhibition of colorectal adenocarcinoma cells [77], while self-assembly has been used to produce nanoparticles that show greater cytotoxic effects to breast cancer cells [78].

While there is a great deal of excitement around the potential applications of sericin in bone grafts [79], there remain gaps and unanswered questions in the current research. The precise mechanism through which sericin influences bone cell growth and the bone healing process is still not entirely understood. Further study into this mechanism can shed light on optimizing the use of sericin in bone grafts. Another critical area for exploration is the long-term biocompatibility of sericin in humans. While in vitro and animal studies suggest sericin is biocompatible [21], there is a need for more comprehensive clinical trials in humans to validate these findings.

Similarly, research into the best methods for incorporating sericin into existing graft materials is ongoing. While there are promising signs from studies using sericin-coated [32] or 3D-printed grafts [64], other techniques could further optimize the use of sericin. Furthermore, given that sericin is derived from silkworms, there is also a need to examine potential allergic reactions or immune responses in patients. Additional research in this area will help ensure the safety and effectiveness of sericin-based bone grafts.

## 6. Conclusions

The potential for sericin, a protein derived from silkworms, as a biomaterial in bone graft applications is evident and promising. With demonstrated advantages such as enhanced biocompatibility, improved osteoinduction, and supportive properties for cell adhesion and proliferation, sericin is gaining increasing attention in the field of tissue engineering. Comparative studies involving sericin-conjugated grafts, particularly silk mat, have shown promising results in bone regeneration, exceeding, or equaling the performance of conventional materials such as expanded polytetrafluoroethylene.

While sericin’s beneficial traits are well recognized, several considerations must be addressed for its optimal application in bone grafting. The choice of scaffold, for instance, plays a critical role, with collagen scaffolds showing superior bone regeneration capabilities when combined with sericin grafts. Further research is warranted to optimize fabrication techniques and scaffold design to maximize the efficacy of sericin-based bone graft materials. The innovative combination of sericin with various synthetic materials, nanostructures, and in mineralization applications demonstrates the versatility of this biopolymer. Notably, the integration of sericin with 3D printing technology opens a new frontier for personalized medicine. The potential to develop patient-specific grafts with enhanced mechanical integrity, degradation properties, and drug delivery applications is significant. Beyond bone graft applications, the sericin-based bioinks have shown promise in the creation of skin substitutes for wound healing and tissue regeneration. Moreover, sericin’s antioxidative, anti-inflammatory, and UV-resistant properties may contribute to a healthier healing environment post-surgery. The deployment of sericin-based nanoformulation in medical applications further expands the scope of this versatile biopolymer.

Despite the promising results to date, gaps in the current research need addressing. The precise mechanism through which sericin influences bone cell growth and healing, the long-term biocompatibility of sericin in humans, potential allergenicity or immune responses, and optimization of sericin incorporation into graft materials are critical areas for future research.

In essence, the versatility, biocompatibility, and potential applications of sericin are promising, heralding a new era of biomaterials in tissue engineering and regenerative medicine. As research progresses, it is envisioned that the use of sericin in bone graft applications will move from the realm of experimental science into routine clinical practice, revolutionizing current tissue engineering strategies.

## Data Availability

No new data were created.
